# A neutral Cu-based MOF for effective quercetin extraction and conversion from natural onion juice†

**DOI:** 10.1039/c9ra04551a

**Published:** 2019-10-21

**Authors:** Rui-Qi Xiang, Yan-Fei Niu, Jie Han, Yat-Long Lau, Hai-Hong Wu, Xiao-Li Zhao

**Affiliations:** Shanghai Key Laboratory of Green Chemistry and Chemical Processes, Department of Chemistry, East China Normal University 3663 North Zhongshan Road Shanghai 200062 P. R. China xlzhao@chem.ecnu.edu.cn +86-21-62233179; School of Science & Technology, The Open University of Hong Kong Kowloon Hong Kong SAR P. R. China

## Abstract

We report herein a new microporous neutral three-dimensional (3D) metal–organic framework [Cu_2_(L)(DMF)(H_2_O)]·*guest* (1·*guest*) composed of copper paddle-wheel and flexible tetracarboxylic acid linkers (DMF = *N*,*N*-dimethylformamide, H_4_L = tetrakis[(6-carboxynaphthoxy)methyl]methane). Surprisingly, this MOF with neutral cavities can not only extract pure quercetin (QT) but also convert it into Cu–QT during the desorption process. It has been well characterized by UV-vis, IR, ESI-MS and TEM-EDS studies. Moreover, it can efficiently extract natural product QT from fresh QT-rich onion juice and rapidly convert it into Cu–QT with a relatively high conversion rate.

## Abstract 2

A new neutral metal–organic framework can efficiently extract natural product quercetin (QT) from fresh QT-rich onion juice and rapidly convert it into Cu–QT with a relatively high conversion rate.

## Graphical abstract

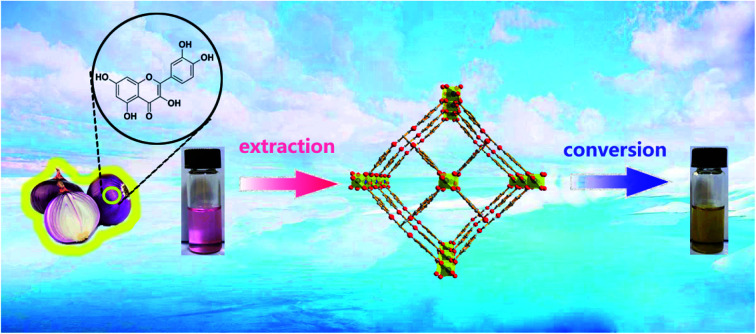

## Introduction

Quercetin (QT, C_15_H_10_O_7_, IUPAC name 2-(3,4-dihydroxyphenyl)-3,5,7-trihydroxy-4*H*-chromen-4-one, see Fig. S1†), a well-known dietary polyphenolic compound, widely exists in a variety of vegetables and fruits, such as onions,^1^ red kidney beans, plums, apples, and black and green teas.^2^ Various pharmacological activities related to quercetin have been documented, including anti-diabetic, anti-oxidant, anti-inflammatory, anti-proliferative, anti-osteoporosis and cancer prevention.^3^ Quercetin is capable of chelating different metal ions, and its copper complex has been demonstrated to exert an improved antioxidant activity and some other biological effects.^4^ Moreover, it is found that the toxicity of copper could be reduced upon its chelation with quercetin, while its bioactivity is maintained.^5^ Due to various pharmacological and biological applications of quercetin and its metal complexes, adsorption and extraction of quercetin from natural resources have been extensively investigated. Porous organic materials, including silica nanoparticles,^6^ polytetrafluoroethylene films,^7^ macroporous acrylic resin^8^ and halloysite,^9^ have been employed in the adsorption and extraction of quercetin. However, most of these materials cannot be effectively recycled. Therefore, the development of recyclable porous materials that can adsorb and extract quercetin with high efficiency has been in great need.

Rapid advances in the field of metal–organic frameworks (MOFs) stem from their intriguing structures, functional diversity and tailorability, that means, the overall structure, pore size and surface functions of a MOF can be fine-tuned through judicious selection of metal nodes, bridging linkers and reaction conditions to satisfy the specific needs of an application.^10^ This designable potential of MOF structures renders them useful for a plethora of applications including gas storage and separation,^11^ heavy metal ions adsorption,^12^ organic pollutants adsorption^13^ and small molecule recognition.^14^ Very surprisingly, despite of the extensive applications of MOFs in adsorption, their uses in natural product extraction have been scarcely reported.^15^

Herein, we demonstrated for the first time that a neutral Cu^II^–MOF [Cu_2_(L)(DMF)(H_2_O)]·*guest* (1·*guest*) (H_4_L = tetrakis[(6-carboxynaphthoxy)methyl]methane) could be used for efficient adsorption of quercetin from both of its artificial solution and a fresh onion juice. Unexpectedly, it was found that quercetin was converted to a Cu–QT complex during desorption. Moreover, such transformation of quercetin to the Cu–QT complex displayed a relatively high conversion rate. Importantly, the Cu–MOF could be readily recovered and reused for several times benefiting from its heterogeneous adsorbent nature.

## Experimental

### Materials and instruments

All the reagents used in this work were commercially available and used as received without further purification. Powder X-ray diffraction (PXRD) patterns were recorded using a Rigaku (D/Max-Ultima IV) diffractometer equipped with Cu Kα radiation (*λ* = 1.54184 Å). Simulated PXRD patterns were calculated with the Mercury program using the single-crystal data.^16^ Thermogravimetric analysis (TGA) was performed using a Netzsch STA449F3 instrument in N_2_ atmosphere in the temperature range of 30–800 °C and a heating rate of 10 °C min^−1^. IR spectra in the range of 500–4000 cm^−1^ was collected using a SHIMADZU IRTracer-100 spectrometer. The ESI-MS was carried out on a Thermo TSQ instrument. UV-vis study was performed on a SHIMADZU UV-2700 UV-vis spectrometer at room temperature. Transmission electron microscope energy-disperse X-ray spectroscopy (TEM-EDS) was conducted on a Hillios G4 UX transmission electron microscope. Crystals of 1·*guest* were evacuated with supercritical CO_2_ in a Tousimis™ Samdri® PVT-30 critical point dryer prior to gas sorption measurements. Low-pressure gases (N_2_ and CO_2_) sorption isotherms (up to 1 atm) were performed on Micromeritics ASAP 2020 surface area.

### Synthesis of [Cu_2_(L)(DMF)(H_2_O)]·*guest* (1·*guest*)

Cu(NO_3_)_2_·2.5H_2_O (0.06 mmol, 13.98 mg), H_4_L (0.04 mmol, 32.68 mg) and nitric acid (50 μL) were added into a mixed solvent (6.0 mL, DMF : H_2_O = 5 : 1) in a 10 mL vial. The mixture was ultrasonicated for 30 min and then kept at 80 °C for three days. After cooling down to room temperature, dark green crystals were isolated by filtration, washed with ethanol and then dried under vacuum at ambient temperature. Yield: 88.74% based on H_4_L. FT-IR (cm^−1^) (Fig. S2†): 2929 w, 2360 vs, 1658 vs, 1481 m, 1384 s, 1257 vs, 1215 vs, 1014 m, 862 s, 777 m, 659 m.

### General activation procedure for 1·*guest*

To obtain the solvent-free material, 5 mg of compound 1·*guest* was immersed in ethanol at ambient temperature for 24 h, subsequently dried at 80 °C for 12 h to yield 1.

### General procedure for adsorption/desorption/conversion of pure QT

The adsorption ability of 1 was evaluated by adsorbing pure QT in ethanol solution at ambient temperature. Typically, 5 mg of activated 1 was soaked in a 2 mL 40 mg L^−1^ quercetin ethanol solution for 24 h. The concentration of QT at a given time was measured by UV-vis spectrophotometer from 200 nm to 500 nm on a SHIMADZU UV-2700 UV-vis spectrometer. Absolute ethanol was used as reference in this experiment. A calibration curve (eqn (S1)†) was plotted to calculate the maximum adsorption amount of QT by 1.

### QT recovery

QT-adsorbed on 1 (QT@1) was immersed into 10 mL of different solvents (acetone, DMF, 2–80% w/v citric acid in ethanol) intending for QT recovery. After immersion for 24 h, the suspension was centrifuged, and the clear solution was measured by UV-vis spectroscopy.

### General procedure for adsorption/desorption/conversion of fresh onion juice

1.0 g of chopped onion was stirred in 6.0 mL ethanol at 60 °C for an hour, and a light purple solution was obtained after filtration. Subsequently, 15 mg of compound 1 was soaked in the obtained solution for 24 h. The conversion behavior of compound 1 toward natural QT was recorded by UV-vis spectroscopy.

### Regeneration of compound 1

To regenerate the compound 1 for the next run, centrifugation followed by washing with ethanol and drying at 80 °C for 12 h was conducted.

### X-ray crystallography

Crystallographic data for 1·*guest* was collected on Bruker Apex duo equipment with Ga radiation (*λ* = 1.34139 Å) at 173 K. The data integration and empirical absorption correction were carried out using SAINT program. Using Olex2 and SHELXTL, the structure was solved by direct method and refined matrix least-squares on *F*^2^ with anisotropic displacement. Non-hydrogen atoms were refined anisotropically, hydrogen atoms were constrained to ideal geometries. The SQUEEZE routine in the program PLATON was used to exclude the contribution from highly disordered solvent molecules that cannot be modeled even with restraints and the SQUEEZE results are appended to the CIF file.^17^ Details of the data collection and crystallographic information are summarized in Table S1.†

## Results and discussion

### Crystal structure and characterizations

**Fig. 1:**
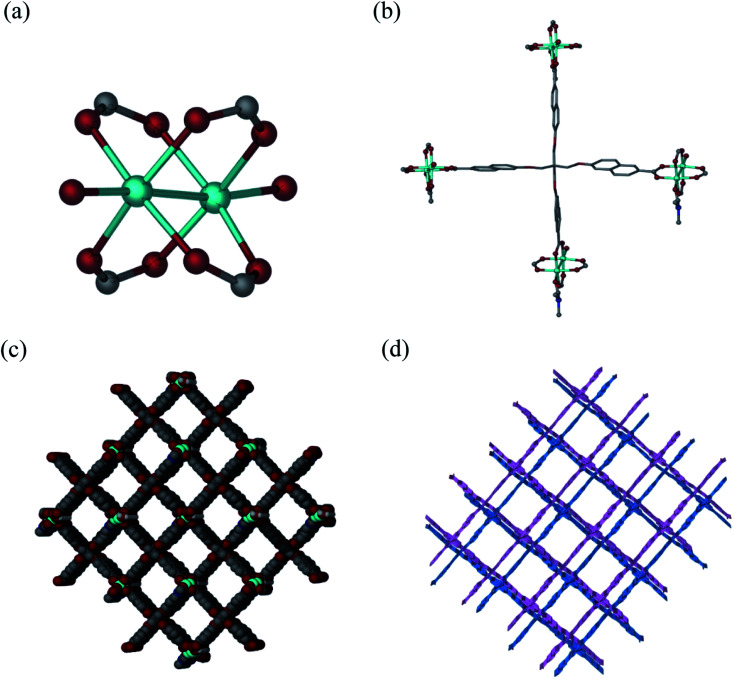
Crystal structure of 1·*guest*. (a) Coordination environment of Cu^2+^; (b) coordination environment around L^4−^; (c) 1D porous channels along the *b*-axis in the 3D framework of 1·*guest*; (d) schematic illustrating the 2-fold interpenetrated structure of 1·*guest*.

Solvothermal reaction of H_4_L and Cu(NO_3_)_2_·2.5H_2_O in a mixture solvents of DMF/H_2_O with the addition of HNO_3_ at 80 °C for 72 h afforded block-shaped crystals of [Cu_2_(L)(DMF)(H_2_O)]·*guest* (1·*guest*). Single-crystal X-ray diffraction analysis reveals that 1·*guest* possesses a 2-fold interpenetrated three-dimensional (3D) (4,4)-connected structure and crystallizes in monoclinic space group *C*2/*c*. The asymmetric unit consists of two Cu^2+^, one L^4−^, one coordinated DMF and an aqua ligand. Paddle-wheel Cu_2_(COO)_4_ SBUs are bridged by L^4−^ to form a 3D net of PtS-type topology, which resembles that of MOF-505 and HKUST-1 (Fig. 1a and b).^18^ Tetragonal channels with the diameter of about 12.7 Å are present when viewed along the *b* axis (Fig. 1c). Due to the existence of large grilles, independent equivalent frameworks further interpenetrated to give a 2-fold interpenetrated architecture as shown in Fig. 1d. PLATON analysis indicates that the solvent accessible volume and porosity are 11 046.5 Å^3^ and 60.7%, respectively. This framework is neutral and the highly disordered solvent molecules reside in the pores.

Powder X-ray diffraction (PXRD) patterns of the as-synthesized 1·*guest* were in good agreement with the simulated ones from the single crystal X-ray diffraction, thus demonstrating the phase purity for 1·*guest* (see Fig. S3†). In addition, PXRD patterns of 1·*guest* upon activation were recorded. The EtOH-exchanged sample was subjected to the activation at 80 °C for 12 h to remove EtOH. PXRD patterns of the activated sample exhibited slight shift to high degree in comparison with the patterns of the as-synthesized sample. It is noteworthy that the crystallinity of 1 still remained. Subsequently, the activated sample was soaked in the mother liquor for another 24 h and the PXRD patterns showed good agreement with the as-synthesized sample. This kind of obvious single-crystal-to-single-crystal transformation demonstrated the flexibility of the framework due to the incorporation of flexible linkers (see Fig. S3 and S4†).

The TGA results revealed that 1·*guest* was stable up to 380 °C under nitrogen atmosphere (see Fig. S5†). Rapid weight loss between 30 and 150 °C prior to decomposition was observed, which corresponded to the release of the entrapped solvent molecules in the pores. When the temperature reached above 380 °C, a sharp weight loss was observed due to the decomposition of the framework.

The N_2_ adsorption of the activated 1·*guest* at 77 K is very low with respect to the calculated porosity (see Fig. S6†), which usually occurs to highly porous MOFs, owing to the distorted structure on removal of the solvent molecules.^19^ While the CO_2_ adsorption measured at 1 atm and different temperatures (273 K, 296 K) are relatively high in comparison with the N_2_ adsorption, revealing excellent selectivity for CO_2_/N_2_. We attribute this to the highly flexible structure involving C–O–C alkyl ether fragments.^20^

### Adsorption and desorption of QT

**Fig. 2:**
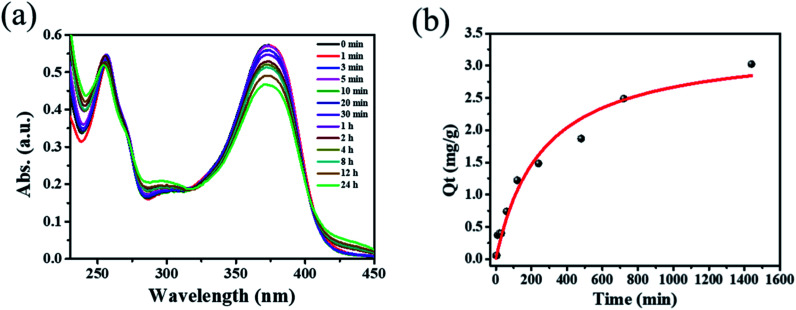
(a) Time dependent UV-vis spectra of 40 mg L^−1^ quercetin ethanol solution with 5 mg of compound 1. (b) Time dependence of the QT adsorption capacity by compound 1.

Inspired by the high porosity and large pores of 1·*guest*, investigations on applications toward large molecule adsorption were subsequently carried out and QT was chosen as a model. The activated 1 (5.0 mg) was immersed into an ethanol solution containing QT (40 mg L^−1^) at room temperature. The adsorption behavior of 1 toward QT was monitored by UV-vis spectroscopy as shown in Fig. 2a. The adsorption capacity for QT by per gram of compound 1 of 3.024 mg g^−1^ was achieved at room temperature in 24 h as shown in Fig. 2b. Taking into account its potential applications for the extraction of natural product QT from fruits and vegetables, the QT-release from QT@1 was then investigated and recorded by UV-vis spectroscopy. Experiments were conducted to optimize the suitable recovery conditions. Aqueous solution of sodium chloride was proven to be not suitable for the desorption experiments in this case due to the instability of compound 1 in the aqueous phase, despite its wide use in the desorption experiments to recovery polyphenolic compounds.^15^ The use of other common polar organic solvents such as acetone and DMF led to a quite low QT recovery rate (<5%) as shown in Fig. S8.† Previously, ethanolic citric acid solution has shown an excellent performance in the recovery of polyphenolic flavonoids with TiO_2_-functionalized mesoporous silica nanoparticles.^6*a*^ Moreover, as a biocompatible ligand with weak acidity, citric acid may help to recover QT from QT@ 1 without destroying structural integrity. Therefore, a range of ethanolic citric acid solution at different concentrations of citric acid were screened. It was found that the structural integrity of 1 was destroyed at 60% w/v concentration of the citric acid as evidenced by PXRD patterns (see Fig. S9†). As a result, 40% w/v ethanolic citric acid was set for the QT recovery (see Fig. S10†). To our surprise, the expected recovery of QT was not observed as monitored by UV-vis spectroscopy (see Fig. S11†). It is known that QT in ethanol solution exhibits two characteristic absorption peaks at 371 nm (band I) and 258 nm (band II), which could be ascribed to ring A (benzoyl system) and ring B (cinnamoyl system) respectively. But the obtained desorption content had a totally different absorption band at 296 nm, which could be assigned to a new material generated from complexation of QT with Cu^II^ according to literature.^21^ The rich hydroxyl and oxo groups in QT dictated its easy complexation with various metal ions. The formation of Cu–QT was further convinced by FT-IR. The characteristic absorptions in IR spectra of QT and the Cu–QT complex in Fig. S12† clearly showed the Cu–QT coordination event. The stretching *ν*(C=O) mode of QT appears at 1664 cm^−1^, which shifts to 1629 cm^−1^ upon coordination with Cu^II^. The formation of Cu–QT complex was further approved by ESI-MS. As shown in Fig. S13,† a peak of *m*/*z* = 482.85 was observed, corresponding to [QT–4H^+^ + 2Cu^II^ + Na^+^]^+^·2H_2_O. Furthermore, EDS was employed to map the distribution of copper ions. The EDS mapping spectrum of compound 1, after desorption at 40% ethanolic citric acid for 24 hours, disclosed the existence of copper element that could be contributed to its chelation with copper ions (see Fig. S14†). Thus, the adsorption of QT by 1 was most likely dominated by its coordination bonding to the SBU of 1.

### QT adsorption/conversion from fresh onion juice

**Fig. 3:**
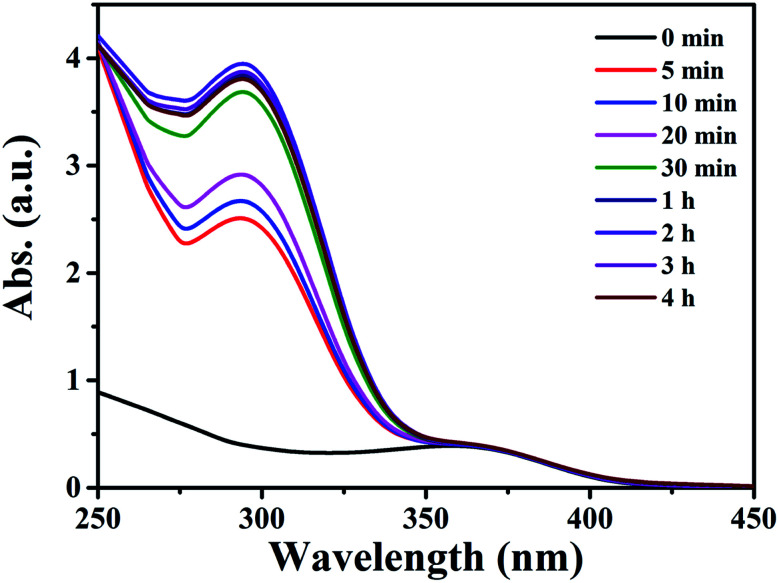
Time dependent UV-vis spectra of 6 mL of an onion ethanol solution with 15 mg of compound 1.

As compound 1 exhibited good adsorption/desorption performance over QT in ethanol, extraction of QT from fresh onion juice (a natural QT-rich material) was thus carried out. As shown in Fig. 3, the maximum absorption peak of QT in fresh onion juice appeared at 360 nm with a slight blue shift,^15^ and a new peak appeared at 298 nm with its intensity increased accompanied by the decrease of the intensity at 360 nm, and no change for both peaks was observed after 24 h. Meanwhile, the color of the fresh onion juice changed from light purple into brownish green, implying the change of the ingredient of the onion juice (see Fig. S15†). In order to identify the origin of the peak at 298 nm, the filtrate was collected and subjected to further characterizations. As shown in Fig. S16,† some characteristic peaks of Cu–QT complex appeared from the IR spectrum of the filtrate, thus providing a direct evidence for the complexation between the copper and natural QT. Moreover, it was confirmed by the observation of mass peak at *m*/*z* = 442.54 that was assigned to the species of [QT–3H^+^ + 2Cu^II^]^+^·H_2_O (Fig. S17†). Additionally, EDS spectrum of the chelating product (Fig. S18†) disclosed the existence of copper element from the filtrate. In this process, compound 1 acted as an intermediary that could provide copper atoms to chelate QT. To the best of our knowledge, there have been very few examples providing the copper atoms of MOFs to chelate the hydroxyl-rich natural products. The main structure of 1 upon first adsorption of QT was demonstrated by the preservation of powder XRD patterns (Fig. S19†).

**Fig. 4:**
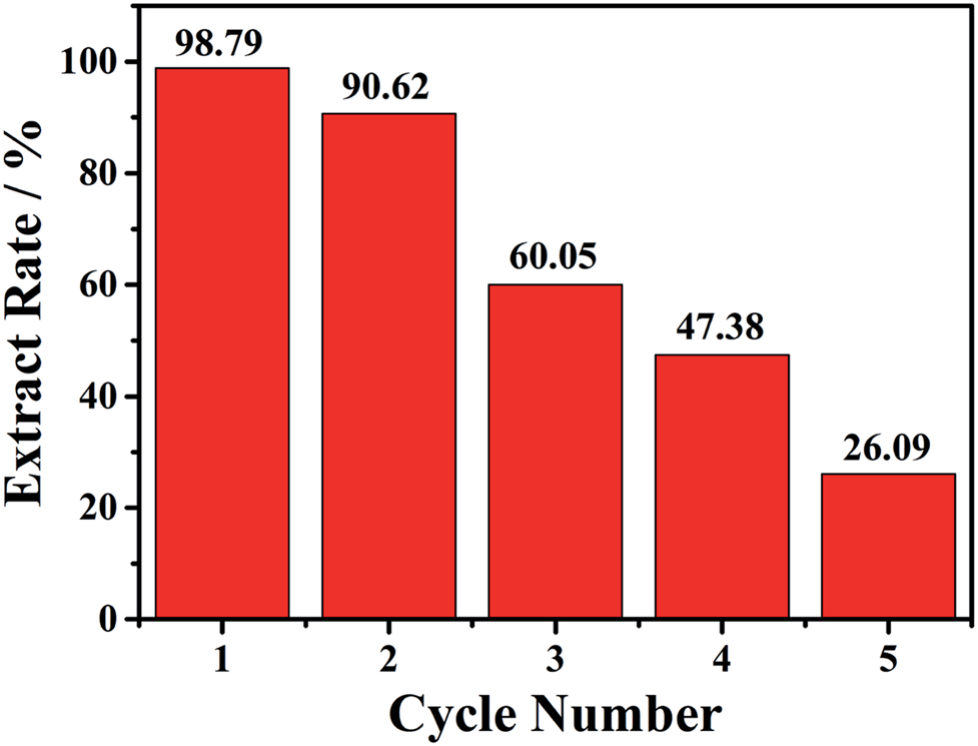
The five cycles of conversion rate of 6 mL onion aqueous solution with 15 mg compound 1 at 30 min.

Then the recycle experiments were conducted. Also, 15 mg of compound 1 was added into 2.0 mL onion solution with a soft shaking for 30 min. After each conversion run, the solid compound 1 was collected, and then added to a new onion solution. As shown in the Fig. 4, the first conversion rate can reach 98.8%, the second conversion rate slightly decreased with the value of 90.6%. Unfortunately, the third, fourth and fifth conversion rates gradually decreased, with the value of 60.1%, 47.4% and 26.1%, respectively. The decreased conversion rate indicated that the loss of copper atoms may affect the structural integrity of 1. PXRD patterns after each conversion exhibited decrease of crystallinity, as shown in the Fig. S19.†

## Conclusion

In summary, a new neutral Cu^II^–MOF with 3D structure has been successfully obtained by assembling of tetracarboxylic acid units with paddle-wheel Cu^II^ SBUs. The synthesized compound 1 can not only adsorb pure quercetin but also convert it into Cu–QT during desorption. Moreover, compound 1 can efficiently extract quercetin from natural onion juice and convert it into Cu–QT with the 98.8% conversion in 30 min. This new finding was an excellent windfall in this process. The results herein could spark potential prospects for loading MOFs which is capable of conversion on absorbent to complete extraction and conversion of medicinal molecules from natural products in one step thus shorten the process.

## Conflicts of interest

There are no conflicts to declare.

## Supplementary Material

RA-009-C9RA04551A-s001

RA-009-C9RA04551A-s002
