# Supplementary material for: A neutral Cu-based MOF for effective quercetin extraction and conversion from natural onion juice
Source: RSC Adv. 2019 Oct 21;9(58):33716–21. doi: 10.1039/c9ra04551a (PMC9073668; doi:10.1039/c9ra04551a)
Supplement: RA-009-C9RA04551A-s001 [file RA-009-C9RA04551A-s001.pdf]

Electronic Supplementary Material (ESI) for RSC Advances.
This journal is © The Royal Society of Chemistry 2019

Supporting Information

A Neutral Cu-Based MOF for Effective Quercetin Extraction and Conversion from Natural Onion Juice

Rui-Qi Xiang,[a] Yan-Fei Niu,[a] Jie Han,[b] Yat-Long Lau,[b] Hai-Hong Wu[a] and Xiao-Li Zhao*[a]

[a]*Shanghai Key Laboratory of Green Chemistry and Chemical Processes, Department of Chemistry, East China Normal University, 3663 North Zhongshan Road, Shanghai 200062, P. R. China.*

[b]*School of Science & Technology, The Open University of Hong Kong, Kowloon, Hong Kong SAR, P. R. China.*

Calculation for quercetin adsorption:

The amount of adsorbed quercetin on compound **1**, q_t (mg quercetin/g compound **1**) was calculated by the depletion method using Equation S1.

$$q_t = \frac{C_0 - C_t}{m} V \times 1000 \qquad \text{(S1)}$$

Where C_0 is the initial concentration (mg/mL); C_t is the concentration of the quercetin solution at any time *t*; and m and V are the mass of compound **1** (mg) and volume of the quercetin solution (in ethanol, mL) used in adsorption experiment, respectively.

Supercritical Drying: MOFs were evacuated with supercritical CO_2 in a Tousimis™ Samdri® PVT-30 critical point dryer. Prior to drying, the DMF/H_2O solvated MOF samples were soaked in ethanol, replacing the soaking solution every 12 hours for 24 hours, to exchange the occluded solvent for ethanol. After the 24 hours, the exchange process was complete and the ethanol-containing samples were placed inside the dryer and the ethanol was exchanged with CO_2 (L) over a period of 1 hour. The rate of venting of CO_2 (L) was always kept below the rate of filling so as to maintain a full drying chamber. After 1 hour of venting and soaking with CO_2 (L), the chamber was sealed and the temperature was raised to around 40 ºC. This brought the chamber pressure to around 1300 psi which is above the critical point of CO_2. The chamber was held above the critical point for 1 hour at which point the chamber was slowly vented over the course of 18-20 hours. The dried samples were used for N_2 and CO_2 adsorptions immediately [1].

[1] A. P. Nelson, O. K. Farha, K. L. Mulfort, and Joseph T. Hupp, *J. Am. Chem. Soc.,*

2009, **131**, 458.

Table S1 Crystallographic data for $[Cu_2(L)(DMF)(H_2O)]$·*guest*

Complex	**1**·*guest*
Empirical Formula	$C_{52}H_{41}Cu_2NO_{14}$
Crystal system	Monoclinic
Space group	*C*2/*c*
a/Å	32.915(4)
b/Å	16.124(2)
c/Å	34.303(4)
α/°	90
β/°	91.696(4)
γ/°	90
V /Å^3	18197(4)
Z	8
ρ_{calcd}. (g cm^{-3})	0.753
μ (mm^{-1})	2.721
F(000)	4240
θ range, °	2.896-56.32
Radiation	GaKα (λ = 1.34139 Å)
R_{int}	0.2043
Final *R* indexes [$I > 2\sigma(I)$]	R_1 = 0.0928,<br>wR_2 = 0.2177
Final *R* indexes [all data]	R_1 = 0.2121,<br>wR_2 = 0.2798

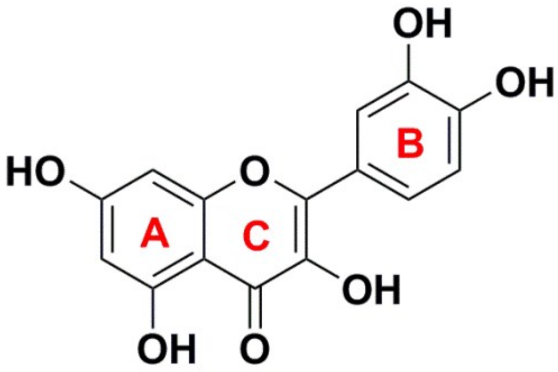

Fig. S1 Chemical structure of quercetin.

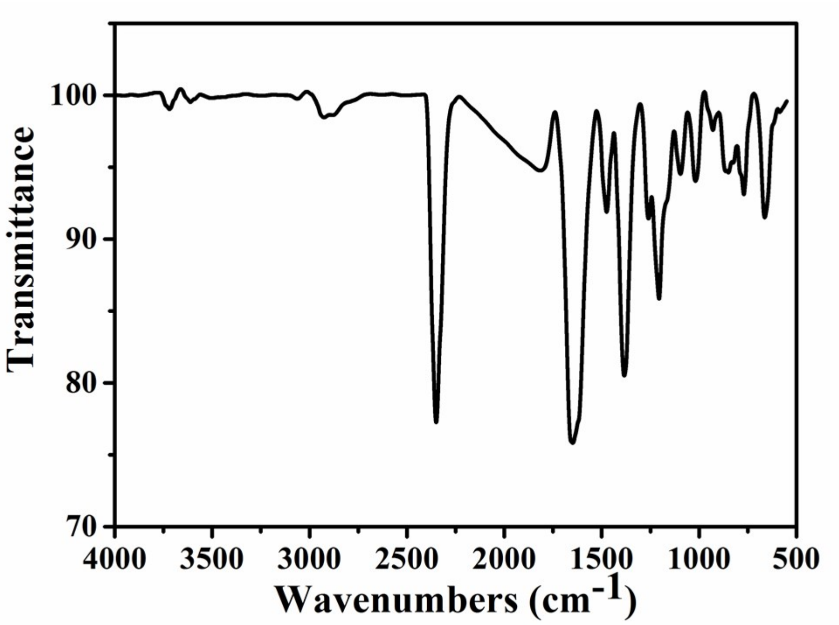

Fig. S2 IR spectra of compound **1**·***guest***.

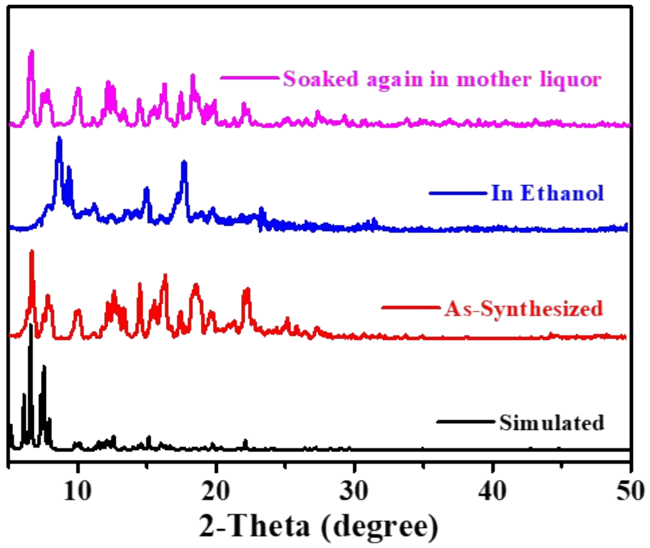

Fig. S3 X-ray powder diffraction patterns for **1**·***guest*** (black, simulated; red, as synthesized; blue, soaked in EtOH for 24h; pink, soaked again in mother liquor for 24 h).

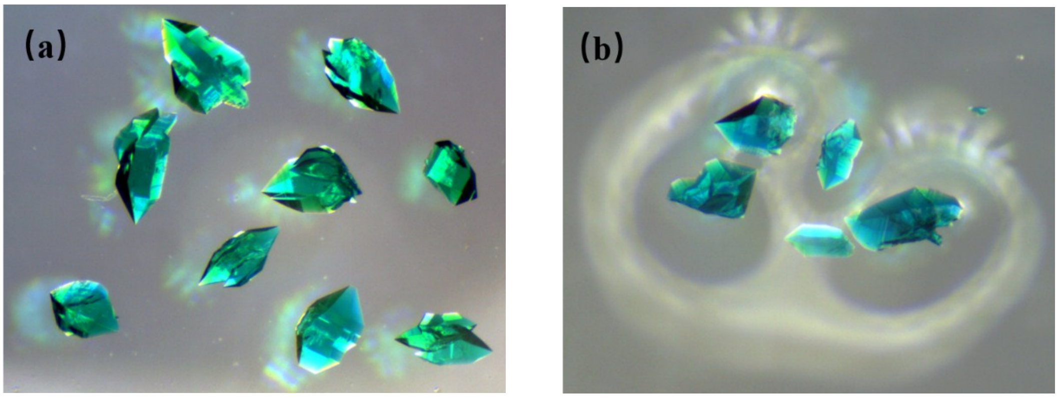

Fig. S4 Photos of crystals. (a) As-synthesized compound **1·*guest***; (b) Immersion in ethanol for 24 h.

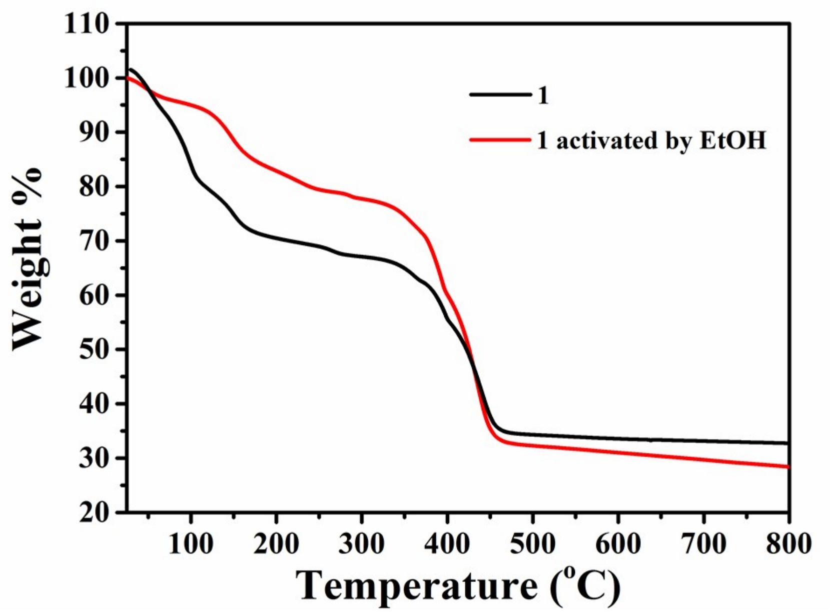

Fig. S5 TGA curves of **1**·***guest*** (black) and EtOH-exchanged (red) sample, respectively.

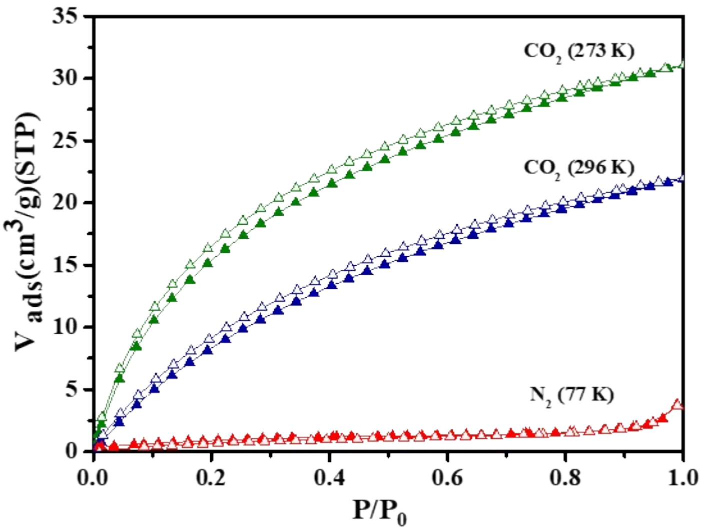

Fig. S6 N_2 and CO_2 sorption isotherms of **1**·***guest***.

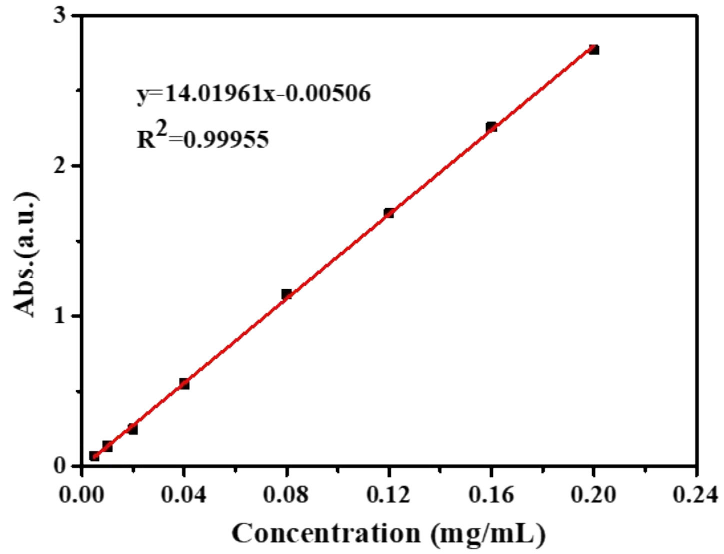

Fig. S7 Standard curve of quercetin in ethanol solution.

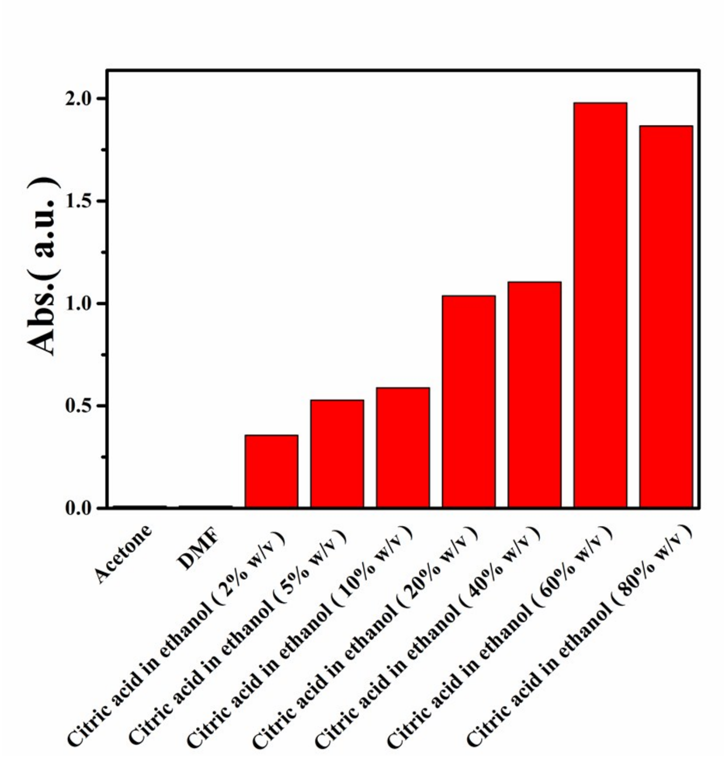

Fig. S8 Recovery of quercetin from **QT@1** using different solvents when 10 mL was used for 15 mg quercetin adsorbed by compound **1**.

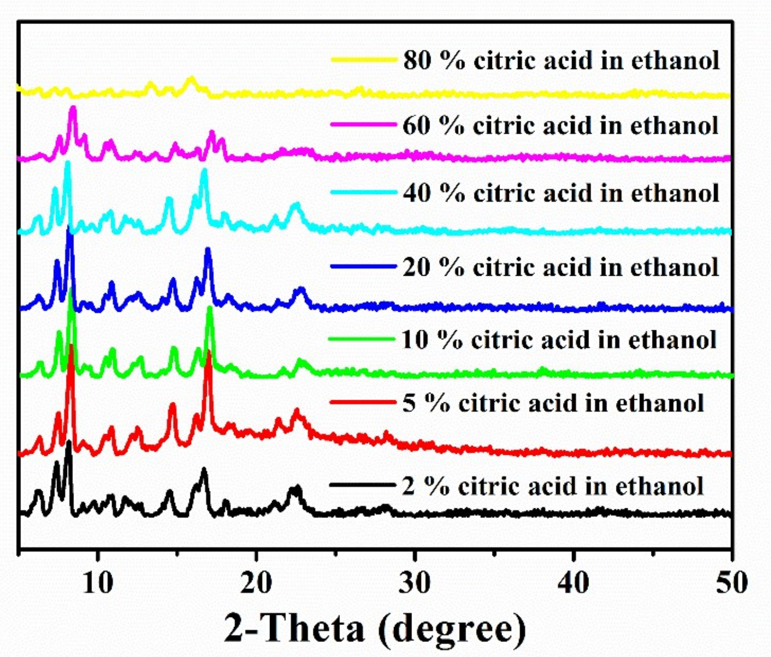

Fig. S9 The PXRD diffraction patterns of QT@1 in a range of ethanolic citric acid solution at different concentrations of citric acid.

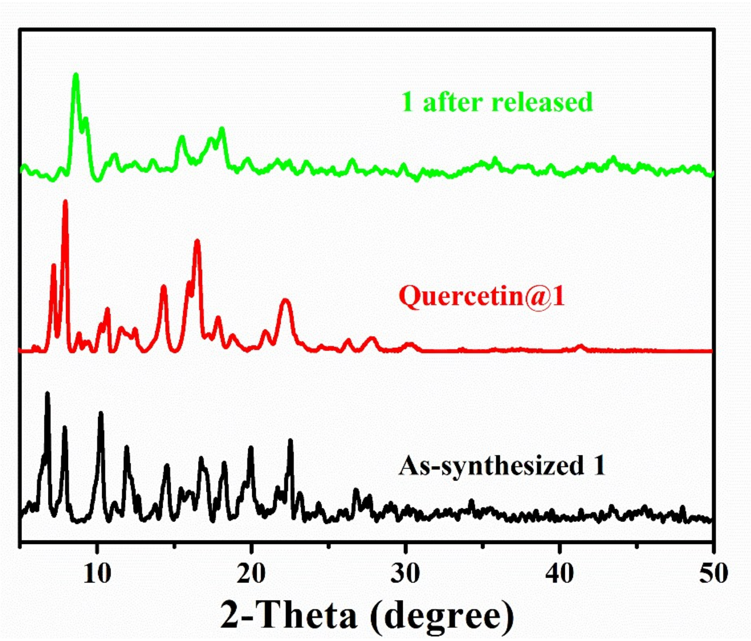

Fig. S10 PXRD diffraction patterns of **1**·***guest***, QT@**1** and QT@**1** in 40% w/v ethanolic citric acid for 24 h.

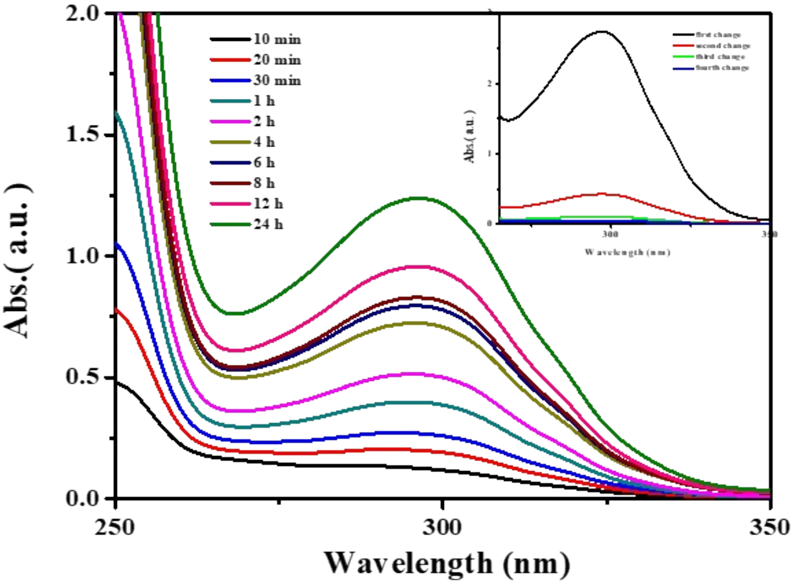

Fig. S11 Time dependent UV-vis spectra of 15 mg of QT @**1** in 50 mL 40% w /v ethanolic citric acid solution; inset: UV-vis spectra of four replacements of 10 mL 40% w/v ethanolic citric acid solution after desorption 24 h.

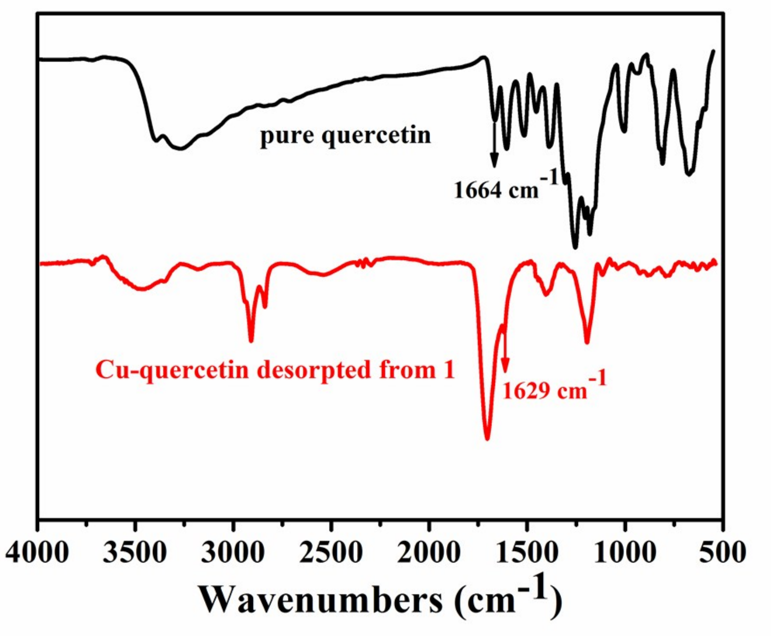

Fig. S12 IR spectra of the Cu-QT from desorption of QT@ **1**.

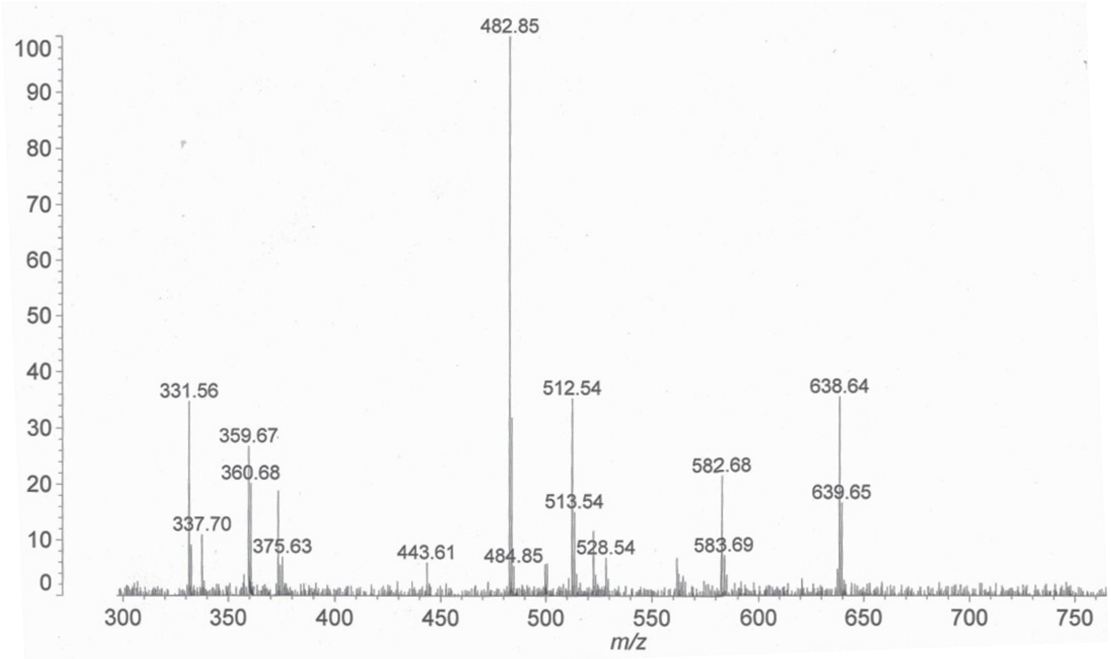

Fig. S13 ESI-MS spectrum for the Cu-QT generated from desorption of QT@ **1**.

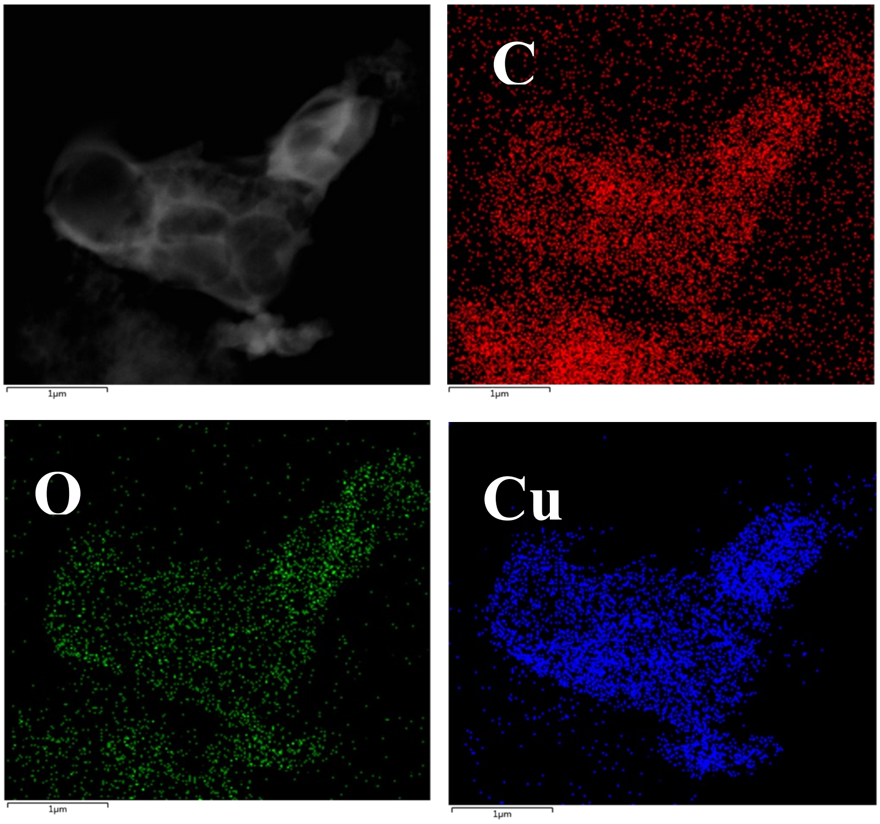

Fig. S14 TEM-EDS mapping of desorption Cu-quercetin.

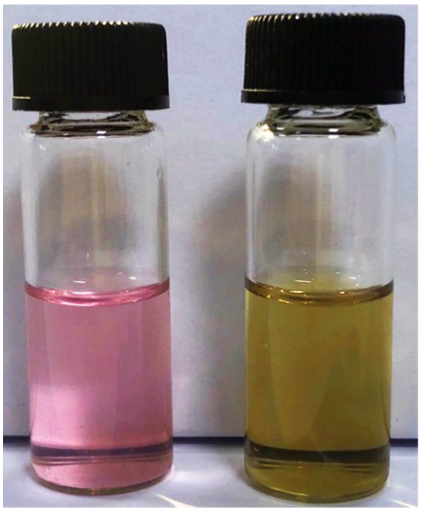

Fig. S15 Color change of the onion solution before and after extraction

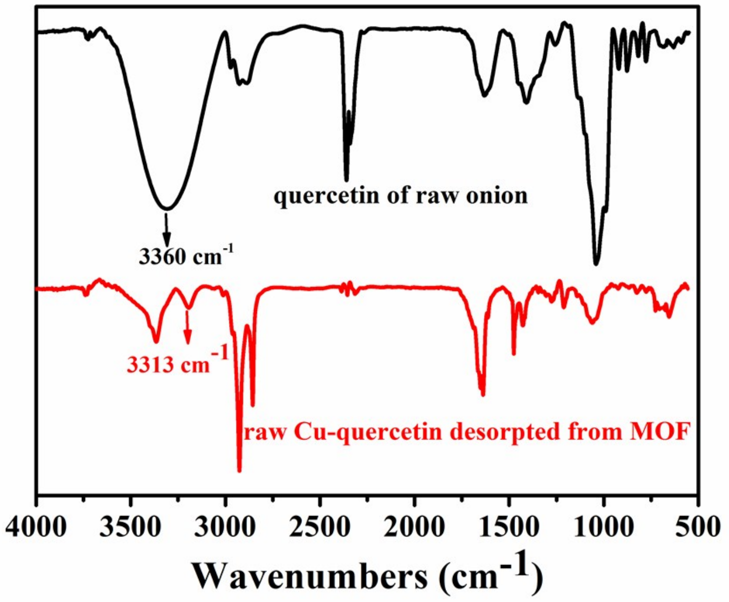

Fig. S16 IR spectrum of the quercetin of raw onion (black) and the natural quercetin conversed in ethanol by compound **1** (red).

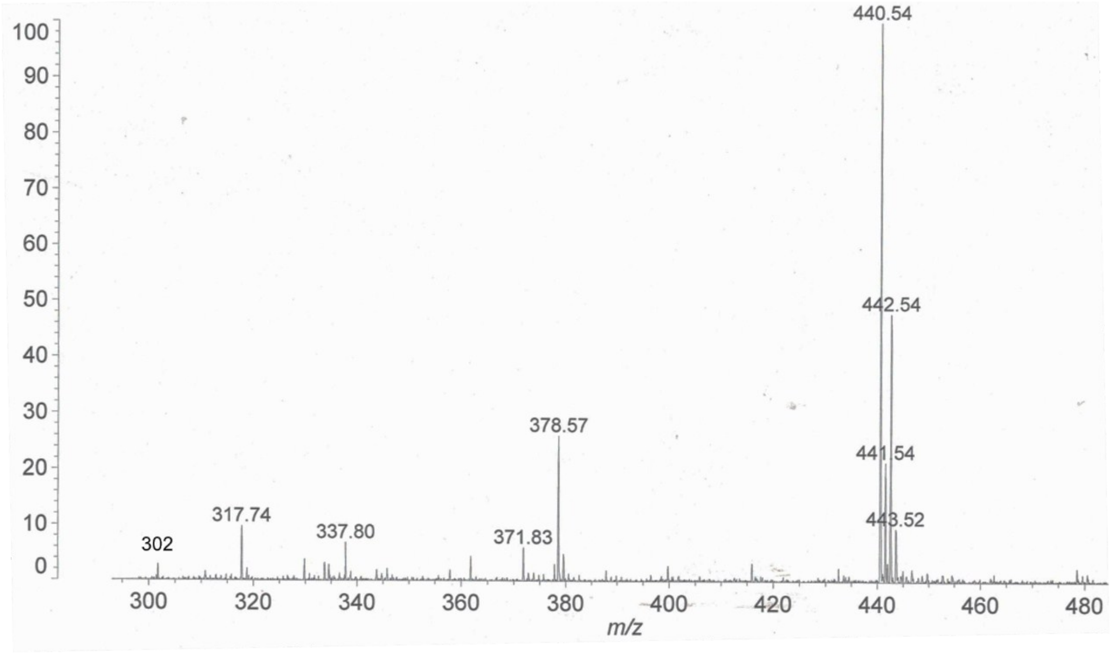

Fig. S17 The ESI-MS spectrum for the filtrate of the natural quercetin conversed in ethanol by compound **1**.

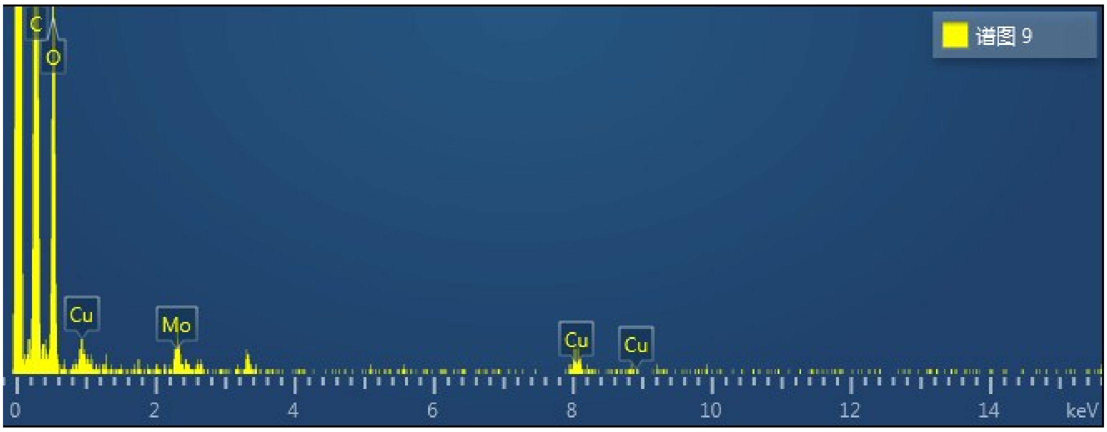

Fig. S18 TEM-EDS spectrum of Cu-natural QT.

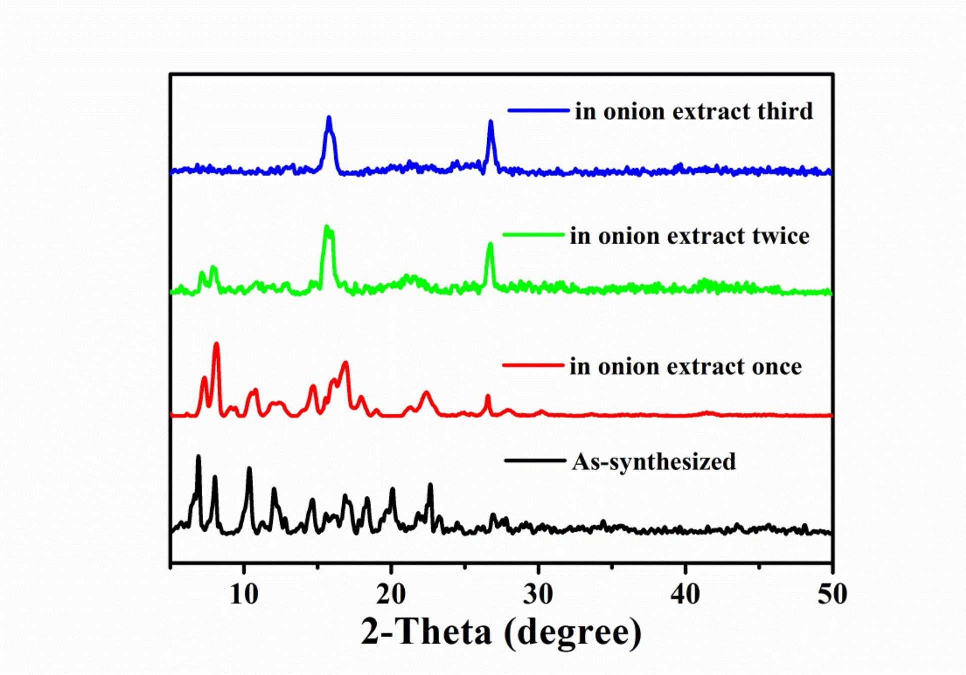

Fig. S19 The PXRD diffraction patterns of **1** in 1-3 runs in onion conversion.

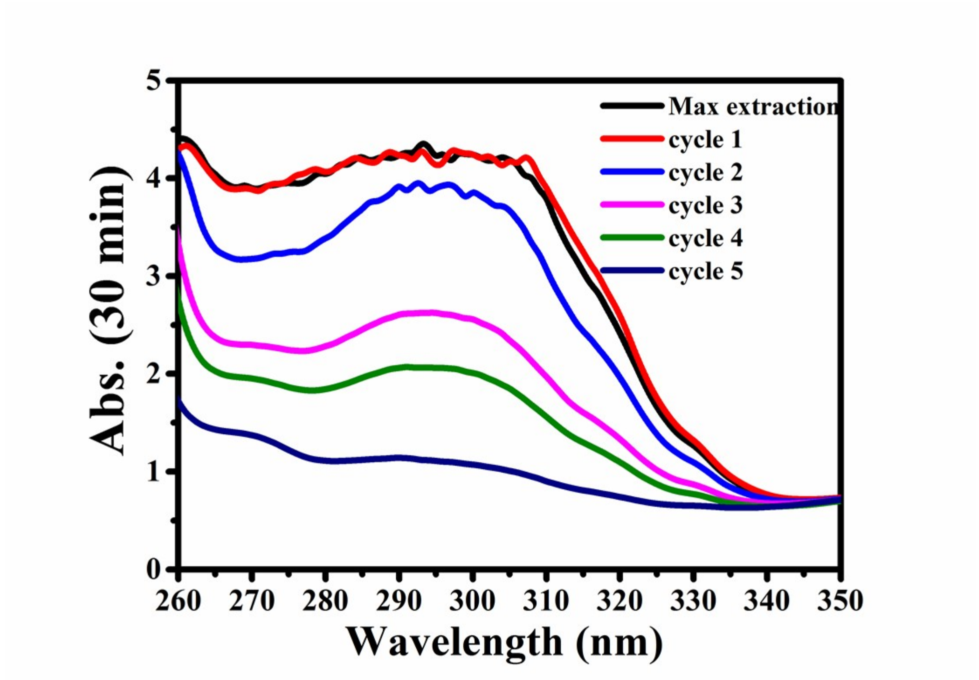

Fig. S20 Time dependence UV-vis spectra for five cycles.

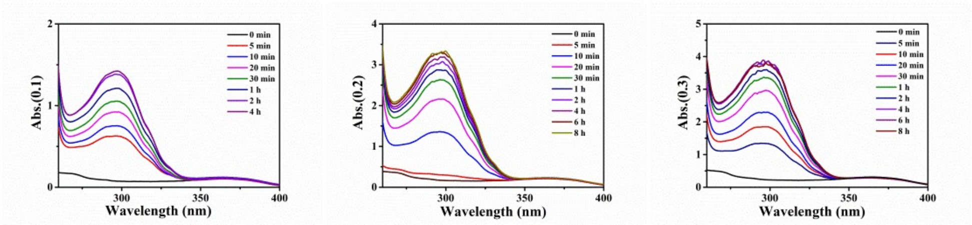

Fig. S21 Time dependence UV-vis spectra of 2 mL onion ethanol solution with the initial absorbance of about 0.1, 0.2, 0.3 with 5 mg **1**.
